# Feasibility for evaluating motor aspects of Parkinson's disease through video consultations in a resource-limited setting in Southern Brazil

**DOI:** 10.1055/s-0043-1768665

**Published:** 2023-05-31

**Authors:** Daniel Teixeira-dos-Santos, Daniel Monte Freire Camelo, Matheus Zschornack Strelow, Maysa Tayane Santos Silva, Paula Führ, Letícia Winer Marins, Artur Francisco Schumacher Schuh

**Affiliations:** 1 Universidade Federal do Rio Grande do Sul, Hospital de Clínicas de Porto Alegre, Serviço de Neurologia, Porto Alegre RS, Brazil.; 2 Universidade Federal do Rio Grande do Sul, Programa de Pós-Graduação em Ciências Médicas, Porto Alegre RS, Brazil.; 3 Universidade Federal do Rio Grande do Sul, Faculdade de Medicina, Porto Alegre RS, Brazil.; 4 Universidade Federal do Rio Grande do Sul, Departamento de Farmacologia, Porto Alegre RS, Brazil.

**Keywords:** Parkinson Disease, Videoconferencing, Telemedicine, Remote Consultation, Developing Countries, Neurologic Examination, Doença de Parkinson, Comunicação por Videoconferência, Telemedicina, Consulta Remota, Países em Desenvolvimento, Exame Neurológico

## Abstract

**Background** Performing motor evaluations using videoconferencing for patients with Parkinson's disease (PD) is safe and feasible. However, the feasibility of these evaluations is not adequately studied in resource-limited settings.

**Objective** To evaluate the feasibility of performing motor evaluations for patients with PD in a resource-limited setting.

**Methods** The examiners rated motor aspects of parkinsonism of 34 patients with PD from the Brazilian public healthcare system through telemedicine with the patient's own means by using the Movement Disorder Society-Sponsored Revision of the Unified Parkinson's Disease Rating Scale (MDS-UPDRS) rating scale. Quality measures of the video meeting were also obtained. The feasibility of rating the motor aspects was the primary outcome whereas the rating of individual motor aspects, video meeting quality and predictors of a complete evaluation served as secondary outcomes.

**Results** The least assessable parameters were freezing of gait (52.9%), gait (70.6%), leg agility, and rest tremor (both 76.5%). Complete MDS-UPDRS part III was possible in 41.2% of patients and 62 out of 374 motor aspects evaluated (16.6%) were missed. Available physical space for a video evaluation was the worst quality measure. Incomplete evaluations were directly associated with disability ( *p* = 0.048, r = 0.34) and inversely with available physical space ( *p* = 0.003, r = 0.55).

**Conclusion** A significant portion of the MDS-UPDRS part III is unable to be performed during telemedicine-based evaluations in a real-life scenario of a resource-limited setting.

## **Resumo**

**Antecedentes** Realizar avaliações motoras usando videoconferência para pacientes com doença de Parkinson (DP) é seguro e viável. Entretanto, a viabilidade dessas avaliações não é adequadamente estudada em cenários com recursos limitados.

**Objetivo** Identificar a viabilidade de realizar avaliações motoras para pacientes com DP em um ambiente com recursos limitados.

**Métodos** Os examinadores avaliaram os aspectos motores da DP de 34 pacientes do sistema público de saúde brasileiro através da telemedicina com os próprios meios do paciente usando a escala *Movement Disorder Society-Sponsored Revision of the Unified Parkinson's Disease Rating Scale* (MDS-UPDRS). Medidas de qualidade da videochamada também foram obtidas. A viabilidade da classificação dos aspectos motores foi o resultado primário, enquanto a classificação dos aspectos motores individuais, a qualidade das videoconferências e os preditores de uma avaliação completa serviram como resultados secundários.

**Resultados** Os parâmetros menos avaliáveis foram congelamento da marcha (52,9%), marcha (70,6%), agilidade dos membros inferiores e tremor de repouso (ambos 76,5%). A parte III completa da MDS-UPDRS foi possível em 41,2% dos pacientes, mas não foi possível avaliar 62 do total de 374 aspectos motores (16,6%). O espaço físico disponível para uma avaliação em vídeo foi a pior medida de qualidade. As avaliações incompletas foram diretamente associadas ao nível de dependência ( *p* = 0,048, r = 0,34) e inversamente ao espaço físico disponível ( *p* = 0,003, r = 0,55).

**Conclusão** Uma porção significativa da parte III da MDS-UPDRS é perdida durante as avaliações baseadas em telemedicina em um cenário da vida real com recursos limitados.

## INTRODUCTION

Patients with Parkinson's disease (PD) need frequent medical consultations and motor assessments to reevaluate the disease's symptoms and adjust treatment. 1 Telemedicine is a promising alternative of care delivery for patients with PD, being a topic of growing interest before the COVID-19 pandemic that was further boosted by it. 2 This modality of care facilitates access to specialists and is associated with reduced travel time, lower financial burden, and higher rate of patient satisfaction, although some technical difficulties and limitations of the neurological examination are also reported. 3

Performing motor evaluations for patients with PD using telemedicine has been previously studied and is considered both safe and feasible. 4 5 6 7 8 Recent studies reported that a successful motor evaluation was possible for 98.3% of the motor parameters present in the Movement Disorder Society – Unified Parkinson's Disease Rating Scale (MDS-UPDRS), except for rigidity and balance, which cannot be assessed in a video conference. 8 Additional research also reported high reliability of the upper limb tele-assessment in comparison to a face-to-face assessment. 9

However, as most studies on the topic of video evaluations for patients with PD were conducted in resource-rich settings and under strict study protocols, 4 5 7 8 the external validity of these findings for developing countries such as Brazil or other resource-limited setting is unknown as educational level and technological limitations can play a role in impeding the implementation of this modality of care. Furthermore, there are few studies on this topic that focus on the low-resource subpopulation, one of which identified that lack of internet connectivity and inability to use technology were the main limitations for the conduction of virtual visits. 10 Additionally, investigating the feasibility of this modality of care in resource-limited settings is essential to better guide the future planning and execution of telemedicine-related clinical trials in this environment, and to reduce disparities in telemedicine application between developing and developed countries.

The present study aims to identify the feasibility of evaluating global and individual motor parameters of patients with PD in follow-up consultations by a movement disorders clinic of the Brazilian public healthcare system in a real-life scenario, as well as to understand possible factors that could be associated with the feasibility of this assessment.

## METHODS

### Design

We conducted a cross-sectional study to address the feasibility of evaluating motor parameters of patients with PD through a video meeting using the technology and software they had available. This study was reviewed and approved by the ethics committee of the Hospital das Clínicas de Porto Alegre. The Strengthening the Reporting of Observational Studies in Epidemiology (STROBE) was followed in the reporting of this study. 11

### Population and data collection procedures

A convenience sample of 35 patients in follow-up by a movement disorders clinic from Brazil's public health system in the Southern region were invited and agreed to participate in the present study. Inclusion criteria were (1) having a hardware such as a smartphone or a computer with a camera, reporting knowing how to use it for a video meeting; and (2) having a diagnosis of PD as per the Movement Disorders Society's diagnostic criteria. 12

Exclusion criteria were being unable to finish the video meeting because of not performing most of the required steps for the motor examination; or having impeditive technological issues, with one patient being excluded due to technical issues related to bad internet connection.

There were six independent examiners with certified training in using the MDS-UPDRS rating scale application, who performed the interview and the video evaluations. Interviewers used their hardware of preference (mostly smartphones and personal computers) to perform the evaluations. Each patient was evaluated by one of the six examiners. All raters ensured they had good technical standards to conduct an adequate motor evaluation during all assessments, such as having a stable and high-quality internet connection, adequate screen and environmental brightness, and sufficient screen size and resolution to observe the patient.

Patients were approached by examiners by telephone calls and a virtual appointment was scheduled based on their availability and best conditions to perform the motor evaluation (e.g., when a patient would have someone available to help them during the evaluation or when they could be present at a location that would facilitate the video evaluation such as the biggest room with the best lighting available).

None of the enrolled patients had previous experience with telemedicine consultations, and all patients were informed of a planned motor evaluation that was similar to the one they performed in the movement disorders clinic they attended.

### Variables

Both sociodemographic and clinical data were collected, including the degree of patient dependency measured by the Schwab and England activities of daily living scale (a 0–100% scale that measures the degree of dependency of patients with parkinsonism). 13 Motor parameters were evaluated using selected items from the MDS-UPDRS motor scale part III, a comprehensive, highly reliable, and widely recognized rating scale that graduates different motor aspects of PD by using a 5-point range system varying from 0 (normal), 1 (slight), 2 (mild), 3 (moderate) to 4 (severe). 14 Permission to use the scale was obtained from the original creators.

The following motor parameters were evaluated: hand movements, leg agility, arising from chair, gait, freezing of gait, global spontaneity of movement, postural tremor of the hands, kinetic tremor of the hands, rest tremor amplitude (excluding lip/jaw tremor), and constancy of rest tremor. These parameters were selected based on their representativeness of the parkinsonian syndrome and expected easiness to evaluate. Rigidity (MDS-UPDRS 3.3) and postural stability (MDS-UPDRS 3.12) were not included as their testing requires maneuvers that need to be performed by a trained rater or cannot be adequately evaluated through video conferencing.

The examiners attempted to rate the selected items as per the scale's standards and deemed specific motor aspects as being unassessable upon meeting certain conditions, such as being unable to distinguish between two subsets of response within the scale (e.g., not being able to differentiate between a normal or a slight rest tremor amplitude), the patient being unable to effectively perform or show the desired motor parameters due to difficulties in comprehending the examiner commands, inadequate positioning of the camera, or other technical limitations. Ultimately, a binary variable regarding if a motor parameter was “ratable” or “unratable” was obtained for each motor aspect. Items with bilateral assessments (such as rest tremor amplitude) were interpreted as unratable if at least one of the evaluations was not possible.

Video meeting quality measures such as internet connection, video, audio and illumination quality, and available physical space (such as having sufficient room for the patient to be completely seen by the camera during examination) were also obtained and rated by the examiner using a Likert scale ranging from 1 to 5, with 1 being very poor, 2 being poor, 3 being regular, 4 being good, and 5 being very good.

### Statistical analyses

The primary outcomes of this study were (1) the possibility of a patient having a complete evaluation (having all 11 selected motor parameters deemed as ratable); and (2) general missed motor aspects (the relation between the number of unratable motor aspects and the total amount aimed to be evaluated).

The secondary outcomes were the ratability of each evaluated motor aspect, quality measures related to the video conferencing, and the association of sociodemographic, clinical, and video meeting quality parameters to having an incomplete evaluation. For this purpose, the primary outcome of having a complete evaluation was binarily divided between “Yes” and “No” and its correlation with variables of interest. Subgroup descriptive analyses were also performed for patients with PD in use of deep brain stimulation.

The Chi-square test was used for categorical variables. For numerical data, the Mann–Whitney U test was performed as distributions were asymmetrical. Effect sizes estimates were further calculated for significant relationships by using the Fisher exact test (Chi-square test) and the rank-biserial correlation (Mann–Whitney U test). Statistical significance was determined by *p-* value ≤ 0.05 and the confidence interval used was 95%.

All analyses were performed using Python (Python Software Foundation, Delaware, USA) version 3.6.9, and the modules Pandas (The PyData Development Team) v. 1.2.5 and SciPy (The SciPy Steering Council) v. 1.7.0.

## RESULTS

### Descriptive statistics

Sociodemographic and clinical data are presented in Table 1 . The mean age was 65.8 years, with 52.9% of the patients being male and 94.1% white. The degree of education and family income were generally low, with only 17.6% having a bachelor's degree or higher and a mean monthly income of 2.7 minimum wages, which is relatively low for meeting basic needs by Brazilian standards.

**Table 1:** Sociodemographic and clinical data

			All patients ( *n* = 34)		DBS patients ( *n* = 10)	
Demographic data	Age		65.8 ± 11.8		58.4 ± 8.8	
	Family income ^a^		2.7 ± 1.3		2.5 ± 1.4	
	Male		18	52.9%	6	60%
	Married or stable union		17	50%	5	50%
	White		32	94.1%	10	100%
	Degree of education	Elementary and middle school or lower	13	38.2%	6	60%
		High school	15	44.1%	4	40%
		Bachelor's degree or higher	6	17.6%	0	0%
Clinical data	Disease's duration (years)		12.8 ± 7.4		18.6 ± 8.5	
	Dementia		6	17.6%	0	0%
	Treatment with deep brain stimulation		10	29.4%	10	100%
	Schwab and England dependency scale (%)		62.6 ± 24.8		65 ± 15.1	
	Used a smartphone for the video consultation		29	85.3%	9	90%
	Used WhatsApp for the video consultation		31	91.2%	10	100%
	Received aid during the video consultation		22	64.7%	7	70%

**Abbreviation:** DBS, deep brain stimulation. **Notes:** ^a^ Measured in monthly minimum wages. Numerical variables displayed with mean ± standard deviation and categorical variables by absolute and relative frequencies.

The mean disease duration was 12.8 years. Furthermore, 17.6% of the patients had concomitant dementia, and 29.4% were in treatment with deep brain stimulation. The mean Schwab and England dependency scale score was 62.6%.

Regarding the means for participating in a video meeting, 86.3% preferred a smartphone, 91.2% used the WhatsApp application for participating, and 64.7% received aid to participate in the procedure. Among patients with deep brain stimulation, the most noticeable differences were a lower mean age (58.4 years), no patients having a bachelor's degree, no concomitant dementia, and a higher disease duration (18.6 years).

### Motor evaluation feasibility and quality measures

**Figure 1:**
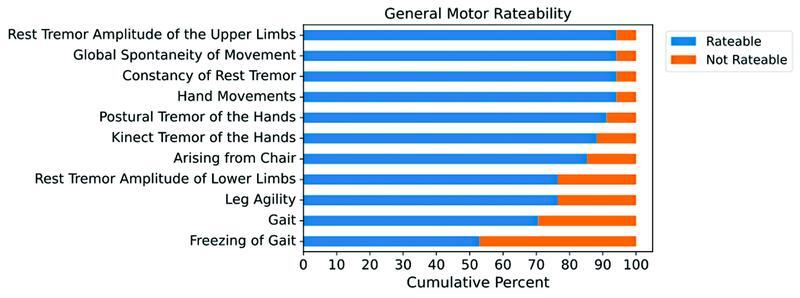
General motor ratability among all patients.

**Figure 2:**
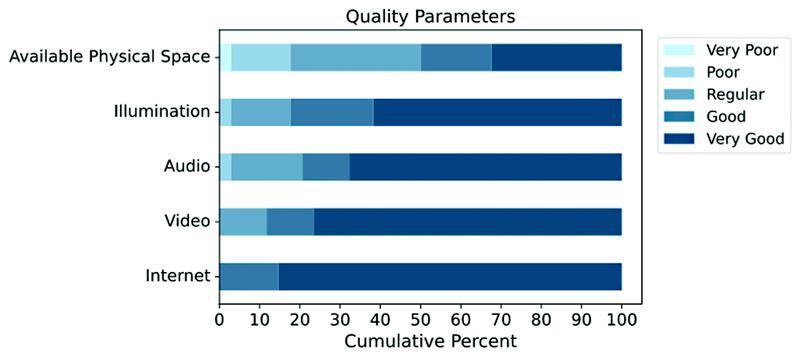
Quality parameters among all patients

Motor parameters evaluation and quality measures of the video consultation are presented in Table 2 . Complete evaluation of all the selected motor parameters was possible in 14 of 34 patients (41.2%). General missed motor aspects were 62 out of 374 items (16.6%). The most commonly ratable parameters were constancy of rest tremor, rest tremor amplitude of the upper limbs, global spontaneity of movement and hand movements (all with a 94.1% ratability). The least assessable parameters were freezing of gait (52.9%), gait (70.6%) and lower limbs leg agility and rest tremor (both 76.5%). The ratability of the motor parameters among all patients is presented in Figure 1 . Aside from available physical space, video consultation quality measures were adequate, with attribute means ranging from 4.4 to 4.9, as measured by a Likert scale – ranging from 1 (very poor) to 5 (very good). Physical space means values were 3.6. A graphical representation of the quality parameters of the video consultation and their response rates among all patients is displayed in Figure 2 . There were no noticeable differences in rating completeness and telemedicine quality measures among patients with deep brain stimulation.

**Table 2:** Motor parameters evaluation feasibility and quality parameters

		All patients ( *n* = 34)		DBS patients ( *n* = 10)	
Motor parameters evaluation completeness	Mean motor parameters evaluated	9.2 ± 2.4		9.6 ± 1.3	
	Complete evaluation (11)	14	41.2%	4	40%
	10	5	14.7%	0	0%
	9	7	20.6%	5	50%
	8	1	2.9%	0	0%
	7 or less	7	20.6%	1	10%
General missed motor aspects		62/374	16.6%	14/110	12.7%
Ratability of motor parameters	Global spontaneity of movement	32	94.1%	10	100%
	Rest tremor amplitude of the upper limbs	32	94.1%	9	90%
	Constancy of rest tremor	32	94.1%	10	100%
	Hand movements	32	94.1%	10	100%
	Postural tremor of the hands	31	91.2%	9	90%
	Kinect tremor of the hands	30	88.2%	10	100%
	Arising from chair	29	85.3%	10	100%
	Leg agility	26	76.5%	7	70%
	Rest tremor amplitude of the lower limbs	26	76.5%	7	70%
	Gait	24	70.6%	8	80%
	Freezing of gait	18	52.9%	6	60%
Quality of the evaluation (Likert scale) ^a^	Internet connection	4.6 ± 0.7		4.6 ± 0.8	
	Video	4.4 ± 0.9		4.5 ± 0.8	
	Audio	4.9 ± 0.4		4.9 ± 0.3	
	Illumination	4.4 ± 0.9		4.4 ± 0.7	
	Available physical space	3.6 ± 1.2		3.5 ± 1.1	

**Abbreviation:** DBS, deep brain stimulation. **Notes:** ^a^ Ranging from 1 (very poor) to 5 (very good). Numerical variables displayed with mean ± standard deviation and categorical variables by absolute and relative frequencies.

### Factors associated with an incomplete evaluation

Correlations between having a complete evaluation and sociodemographic, clinical, and quality measures are presented in Table 3 . Having an incomplete evaluation was associated with higher scores in the Schwab and England dependency scale ( *p* = 0.048, r = 0.34) and lower available physical space quality ( *p* = 0.003, r = 0.55). The r values ranging from 0.2 to 0.49 and from 0.5 to 0.79 are considered weak and moderate correlation, respectively. 15 Deep brain stimulation treatment did not influence motor evaluation completeness.

**Table 3:** Predictors of a complete evaluation among all patients

	Had a complete evaluation?		*p* -value	Effect size
	Yes ( *n* = 14)	No ( *n* = 20)		
Age	65.9 ± 11.0	65.8 ± 12.1	0.989	−
Male	9 (64.3%)	9 (45.0%)	0.447	−
Family income ^a^	2.4 ± 1.1	2.9 ± 1.4	0.218	−
Disease's duration (years)	11.6 ± 9.8	13.6 ± 4.6	0.053	−
Elementary and middle school or lower	5 (35.7%)	8 (40.0%)	0.886	−
Dementia	1 (7.1%)	5 (25.0%)	0.375	−
Treatment with deep brain stimulation	4 (28.6%)	6 (30.0%)	0.77	−
Received aid during the video consultation	9 (64.3%)	13 (65.0%)	0.748	−
Schwab and England dependency scale	72.1 ± 20.1	56.0 ± 25.0	**0.048**	**0.34**
Internet connection quality	4.7 ± 0.6	4.6 ± 0.7	0.38	−
Video quality	4.6 ± 0.9	4.3 ± 0.9	0.171	−
Audio quality	4.9 ± 0.3	4.7 ± 0.6	0.09	−
Illumination quality	4.6 ± 0.6	4.3 ± 1.0	0.279	−
Available physical space quality	4.3 ± 1.0	3.1 ± 1.1	**0.003**	**0.55**

**Notes:**^a^ Measured in monthly minimum wages. Numerical variables displayed with mean ± standard deviation. Effect sizes reported only for significant relations (Rank-biserial correlation). Bold numbers indicate statistical significance.

## DISCUSSION

This study aimed to evaluate the feasibility of assessing pivotal motor parameters of patients with PD in a real-life scenario with limited resources, as well as possible factors associated with an incomplete evaluation. We identified that a successful complete motor evaluation of important aspects of parkinsonism (including limb bradykinesia, rest, postural and action tremors, arising from chair, gait, and freezing of gait) was low. The number of unevaluated motor aspects were also significant, being almost ⅕ of the proposed motor parameters. Motor aspects pertaining to gait, lower limb bradykinesia, and rest tremor were the least ratable, while motor parameters related to upper limb tremor and bradykinesia were the most ratable. To the best of our knowledge, this is one of the few studies to assess the feasibility of evaluating motor characteristics in patients with PD through a video meeting in a real-life scenario in a setting with limited resources.

In contrast to our findings, previous studies on this topic reported better feasibility in evaluating motor aspects of patients with PD by using different variables and designs. A recent study conducted in China with patients that underwent deep brain stimulation surgery reported that 18 out of 22 patients (81.8%) were able to complete the motor assessment of a modified MDS-UPDRS scale. 16 Schneider et al. 8 reported that it was feasible to rate 98.3% of all the MDS-UPDRS motor parameters (except rigidity and postural instability) among approximately 550 patients that were previously enrolled in clinical trials studies for PD. Finally, Stillerova et al. 17 evaluated 11 Australian patients with PD in a real-life scenario by using 18 motor aspects present in the MDS-UPDRS and reported a median of 2 motor items missing for each patient (11.1% of the proposed motor aspects). Other studies also reported high rates of completeness of motor evaluations through video conferencing, either by using a modified version of the MDS-UPDRS that excluded rigidity and postural stability in highly selected populations, 4 5 6 7 or other instruments pertaining to upper limb functioning. 9

There are a variety of reasons for difficulties in assessing motor parameters and for the difference observed between the present and previous studies. First, adequately performing a videoconference evaluation a patient needs both technical and patient-related factors to be present. In addition, patients need to have access to adequate hardware and software that can permit the video call to proceed with the most desirable image quality and frame ratio, the internet connection must be stable and of minimum speed, and patients must be in a suitable room regarding physical space, camera mobility, room brightness, among other factors. Additionally, patients or caretakers need to be instructed in how to use these technologies and be able to follow the examiner's instructions appropriately. Most studies reported that at least some of these components impaired adequate motor evaluations. 6 8 10 17 Studies in which these difficulties were not reported most often offered technical support and instructions, and had highly selected populations regarding their educational level and ease to use this technology. 4 5

Due to our study being conducted in a real-life scenario of a resource-limited country, most patients had a low degree of education and income, which is possibly associated with a lower quality of hardware and internet connection available for a video meeting, as well as more difficulties in dealing with technology and understanding the examiner's instructions. The patients' available physical space was the worst-ranked quality parameter, which can be explained by socioeconomic status. Additionally, our population had a longer disease duration and was more dependent than those enrolled in other studies, 5 which are possible factors that could have impeded adequate evaluations. This is further supported by our findings that these characteristics were inversely associated with the feasibility of a complete motor evaluation through a video meeting. In conjunction, these demographic and clinical factors probably played a role in hindering complete and individual motor evaluations in our research compared with others. Lastly, performing the video consultation with the help of a caretaker did not affect the motor evaluation completeness, which could indicate that difficulties in camera positioning or in following commands due to severe motor symptoms were not a prominent predictor of the evaluation's success.

We identified that the least ratable parameters were associated with the evaluation of lower limbs and gait. Incomplete evaluation of lower limbs tremor and bradykinesia was also reported in other studies, 8 17 but only the research conducted by Stillerova et al. also reported gait and freezing of gait evaluation as difficult. 17 Taken together, these observations indicate that factors such as physical space for a video examination are the most important components in restraining an adequate examination. This interpretation is further corroborated by the observation that a small physical space was significantly associated with an incomplete motor evaluation in our study.

A limitation of this study is that not all possible motor aspects of the MDS-UPDRS part III were evaluated, and that no face-to-face interviews were performed to compare our findings to the ones in the video meeting. However, the aim of our study was not to contribute to the validation of the MDS-UPDRS for telemedicine, a topic currently under study, 18 but to use examples of motor aspects deemed as important and representative of parkinsonism that could be scored in a strict and already consolidated rating scale. Additionally, a higher rate of missing motor evaluations can be attributable to unfamiliarity in rating clinical aspects of parkinsonism through telemedicine, even though examiners had training in using the rating scale. External validity of our findings may differ across other outpatient settings, as a great portion of our patients had longer disease duration and more clinical dependency (denoted by a high mean disease duration and low mean Schwab and England dependency score). Lastly, the limited sample size may hinder the results' interpretation and generalization.

The advantages of this study were it being the first to address this topic in a real-life and resource-limited setting, and reporting difficulties in rating motor evaluation using different variables (e.g., complete evaluations and general missing motor aspects). Also, this is one of the few studies to evaluate the relationship between demographic, clinical, and quality parameters, and having a complete motor evaluation.

Even though the feasibility for complete motor evaluations through telemedicine among our studied population was limited, it is noteworthy that a significant number of patients had an almost complete motor evaluation. To better improve evaluation quality and completeness, healthcare providers could organize strategies for implementation of telemedicine, such as the creation of satellite clinics and raising general awareness on this modality of consultation. Ultimately, these protocols could also prove to be cost-effective due to a reduced commute time and easier healthcare access, especially for patients that live in remote or rural areas. Failure to address these topics and better provide telemedicine services to patients with movement disorders may exacerbate the already existing disparities in access to healthcare among countries, as previously appointed by a global survey on telemedicine. 19

In conclusion, motor evaluations of pivotal aspects of PD through a video meeting in a real-life scenario with limited resources face many limitations and often missed rating of different motor parameters, although still viable for some patients. Motor aspects pertaining freezing of gait, lower limbs bradykinesia, and rest tremor were the least ratable. The available physical space was the worst quality parameter. Furthermore, higher degree of dependency and lower available physical space for the video consultation were associated with an incomplete motor evaluation. Telemedicine for patients with PD facilitates access to specialists and is a highly satisfactory and cost-effective way. Its implementation in resource-limited settings needs to acknowledge and overcome these reported limitations on performing motor evaluations.

## References

[1] Little M Wicks P Vaughan T Pentland A Quantifying short-term dynamics of Parkinson's disease using self-reported symptom data from an Internet social network J Med Internet Res 2013 15 01 e20 10.2196/jmir.2112 23343503 PMC3636067

[2] MDS-Scientific Issues Committee Papa S M Brundin P Fung V SC Impact of the COVID-19 Pandemic on Parkinson's Disease and Movement Disorders Mov Disord 2020 35 05 711 715 10.1002/mds.28067 32250460 PMC7996401

[3] van den Bergh R Bloem B R Meinders M J Evers L JW The state of telemedicine for persons with Parkinson's disease Curr Opin Neurol 2021 34 04 589 597 33990100 10.1097/WCO.0000000000000953 PMC8279892

[4] Dorsey E R Wagner J D Bull M T Feasibility of Virtual Research Visits in Fox Trial Finder J Parkinsons Dis 2015 5 03 505 515 26406130 10.3233/JPD-150549 PMC4923707

[5] Tarolli C G Andrzejewski K Zimmerman G A Feasibility, Reliability, and Value of Remote Video-Based Trial Visits in Parkinson's Disease J Parkinsons Dis 2020 10 04 1779 1786 10.3233/jpd-202163 32894251

[6] Randall A Panicker J Macerollo A Alusi S H To assess whether a “virtual admission” can be useful for Parkinson's disease patients with severe motor fluctuations Neurol Sci 2021 42 06 2543 2545 10.1007/s10072-020-04939-9 33409827 PMC7787934

[7] Sekimoto S Oyama G Hatano T A Randomized Crossover Pilot Study of Telemedicine Delivered via iPads in Parkinson's Disease Parkinsons Dis 2019 2019 9.403295E6 10.1155/2019/9403295 PMC6339724 30723541

[8] Schneider R B Myers T L Tarolli C G Remote Administration of the MDS-UPDRS in the Time of COVID-19 and Beyond J Parkinsons Dis 2020 10 04 1379 1382 32675421 10.3233/JPD-202121 PMC12803732

[9] Cabrera-Martos I Ortiz-Rubio A Torres-Sánchez I López-López L Rodríguez-Torres J Carmen Valenza M Agreement Between Face-to-Face and Tele-assessment of Upper Limb Functioning in Patients with Parkinson Disease PM R 2019 11 06 590 596 30840363 10.1002/pmrj.12001

[10] Shalash A Fathy M Dawood N L Hamid E Adopting Virtual Visits for Parkinson's Disease Patients During the COVID-19 Pandemic in a Developing Country Front Neurol 2020 11 582613 10.3389/fneur.2020.582613 33193042 PMC7652791

[11] STROBE Initiative von Elm E Altman D G Egger M Pocock S J Gøtzsche P C Vandenbroucke J P The Strengthening the Reporting of Observational Studies in Epidemiology (STROBE) statement: guidelines for reporting observational studies Bull World Health Organ 2007 85 11 867 872 10.2471/blt.07.045120 18038077 PMC2636253

[12] Postuma R B Berg D Stern M MDS clinical diagnostic criteria for Parkinson's disease Mov Disord 2015 30 12 1591 1601 26474316 10.1002/mds.26424

[13] Bjornestad A Tysnes O B Larsen J P Alves G Reliability of Three Disability Scales for Detection of Independence Loss in Parkinson's Disease Parkinsons Dis 2016 2016 1.941034E6 10.1155/2016/1941034 PMC4860247 27213080

[14] Movement Disorder Society UPDRS Revision Task Force Goetz C G Tilley B C Shaftman S R Movement Disorder Society-sponsored revision of the Unified Parkinson's Disease Rating Scale (MDS-UPDRS): scale presentation and clinimetric testing results Mov Disord 2008 23 15 2129 2170 19025984 10.1002/mds.22340

[15] Khamis H Measures of Association: How to Choose? J Diagn Med Sonogr 2008 24 03 155 162 10.1177/8756479308317006

[16] Xu X Zeng Z Qi Y Remote video-based outcome measures of patients with Parkinson's disease after deep brain stimulation using smartphones: a pilot study Neurosurg Focus 2021 51 05 E2 10.3171/2021.8.FOCUS21383 34724646

[17] Stillerova T Liddle J Gustafsson L Lamont R Silburn P Remotely Assessing Symptoms of Parkinson's Disease Using Videoconferencing: A Feasibility Study Neurol Res Int 2016 2016 4.80257E6 10.1155/2016/4802570 PMC5220499 28116158

[18] Parkinson Study Group AT-HOME PD Investigators Schneider R B Omberg L Macklin E A Design of a virtual longitudinal observational study in Parkinson's disease (AT-HOME PD) Ann Clin Transl Neurol 2021 8 02 308 320 33350601 10.1002/acn3.51236 PMC7886038

[19] International Telemedicine Study Group Hassan A Mari Z Gatto E M Global Survey on Telemedicine Utilization for Movement Disorders During the COVID-19 Pandemic Mov Disord 2020 35 10 1701 1711 32833273 10.1002/mds.28284 PMC7461376

